# An empirical study on the usage behavior of mobile health management service systems for flight attendants in the digital Age

**DOI:** 10.3389/fdgth.2025.1497549

**Published:** 2025-04-14

**Authors:** Yuting Liu, Haiyan Li

**Affiliations:** ^1^ Civil Aviation Security College & Physical Education Department, Civil Aviation Flight University of China, Guanghan, China; ^2^ School of Educational Studies, Universiti Sains Malaysia, Penang, Malaysia

**Keywords:** digital age, flight attendants, mobile health management services, usage behavior, structural equation model

## Abstract

**Objective:**

Mobile health management service systems have rapidly emerged in today's digital age, providing a new way to manage personal health with great potential value. This study deeply explores the use behavior and influencing factors of mobile health management service systems for flight attendants in the context of the "Digital age".

**Methods:**

The study mainly adopted the questionnaire survey method, used SPSS24.0 and AMOS24.0 software for data analysis, and used statistical methods such as factor analysis, regression analysis, and path analysis to verify the effectiveness of the model and explore the relationship between key variables.

**Results:**

(1) The usage rate of the health management service system among flight attendants is not as high as expected, but the use rate of sports health monitoring applications reached 66.5%, and the daily frequency of use was as high as 25.52%, (2) Perceived ease of use and perceived usefulness have a positive and significant impact on intention to use, (3) Privacy concerns have a positive impact on intention to use Significant negative impact.

**Conclusion:**

The study points out that ease of use and usefulness are key factors in attracting flight attendants to use mobile health management service systems. System designers need to pay attention to this aspect. Flight attendants have a strong sense of privacy protection, and the system must provide a strong privacy protection mechanism to win trust. Therefore, system developers should strive to provide practical value, such as health advice and data tracking, to stimulate user enthusiasm. In addition, this article has certain limitations in the study of sample selection and the discussion of mediating relationships. Future research can be further improved in this regard.

## 1 Introduction

The core of the "Healthy China 2030" Plan is to focus on people's health and adhere to the grassroots (1). Flight attendants are grassroots service personnel working in the civil aviation industry. Due to high-altitude hypoxia and radiation, noise pollution, excessive workload, special dress requirements, maintaining good appearance and demeanor, strict work regulations, disordered work and rest time, changes in physiological rhythms, and high-risk flight industry stress, flight attendants have increased physical load and increased morbidity (2). In 2019, the State Council's “Opinions on Implementing the Healthy China Action Plan” proposed the implementation of occupational health protection actions, and workers have the right to occupational health protection according to law. For different occupational groups, healthy working methods are advocated, and the prevention and control of occupational hazards are emphasized (3). Therefore, the health management of flight attendants is particularly important both from the perspective of policy advocacy and practical work needs. The continuous development and maturity of emerging technologies such as cloud computing, big data, the Internet of Things, mobile Internet, and artificial intelligence have accelerated the integration of the traditional medical industry with emerging technologies of new infrastructure. New medical formats represented by health and medical big data, smart medicine, and Internet + medicine have emerged, which continue to promote the development of the medical industry (4–6). In addition, the “Guiding Opinions on Promoting and Regulating the Development of Health and Medical Big Data Applications” in June 2016 also mentioned the need to follow the development trend of emerging information technology and regulate and promote the integration, sharing and open application of health and medical big data (7). According to the 53rd “Statistical Report on the Development of China's Internet”, as of December 2023, the scale of Internet users in my country reached 1.092 billion, with an increase of 24.8 million Internet users compared to December 2022, and the Internet penetration rate reached 77.5%. The scale of Internet medical users in my country reached 414 million, and the scale of online medical users in my country reached 239 million, accounting for 23.7% of the total number of Internet users (8). With the in-depth development of 5G and mobile Internet, a mobile phone, an iPad, or a computer can easily solve daily life problems, including chronic disease management, registration and consultation, online pharmacies, doctor-patient communication, and other health-related fields (9). With the popularization of mobile Internet and smart phones, mobile health management services have become a model with great development potential, and have achieved results in many aspects of the health field, and have been widely concerned, accepted and loved by the public. However, at present, my country's mobile health management services are still in the exploratory stage. Mobile health management services are relatively simple and elementary, and the homogeneity problem in the industry is prominent. In addition, there is a lack of sufficient understanding of the potential needs of specific user groups, and user acceptance and satisfaction need to be improved (10). Therefore, this topic builds a model based on the technology acceptance model theory and social cognition theory for the user group of flight attendants to explore the promoting factors and hindering factors that affect the use behavior of mobile health management service systems. This is not only theoretically innovative, but also enriches Relevant theoretical knowledge also has practical guiding significance for flight attendants to prevent and control occupational diseases and achieve self-health management with knowability, visibility, and reliability.

## 2 Theoretical basis and research hypothesis

### 2.1 Concept of mobile health management services

The mobile health management service system is a digital platform that uses mobile communication technology and information technology to provide individuals with health management, medical services, and health information. This system aims to help users more conveniently conduct health monitoring, disease prevention, health education, and medical services through mobile applications, smart devices, and Internet technology (11). Based on the existing data, this paper divides the mobile health management service system into the following specific categories (see Table 1).

**Table 1:** Categories of mobile health management service systems.

Category	Specific name
Diet	Mint, Food Pie, Healthy Recipes, etc.
Sleep	Little Sleep, Dolphin Sleep, TING, etc.
Blood Sugar	Blood Sugar Executive, Sugar Nurse, Micro Sugar, etc.
Blood pressure category	Blood pressure record, Blood pressure manager, Kang Kang blood pressure, etc.
Body fat health category	Slim, Meite, Phicomm Health, Daily Yoga, etc.
Sports and fitness category	Keep, Nike+, Xiaomi Sports, Huawei Health Sports, etc.
Applications embedded in the APP	WeChat Sports, Alipay Sports, QQ Sports, etc.

### 2.2 Technology acceptance model theory

**Figure 1:**
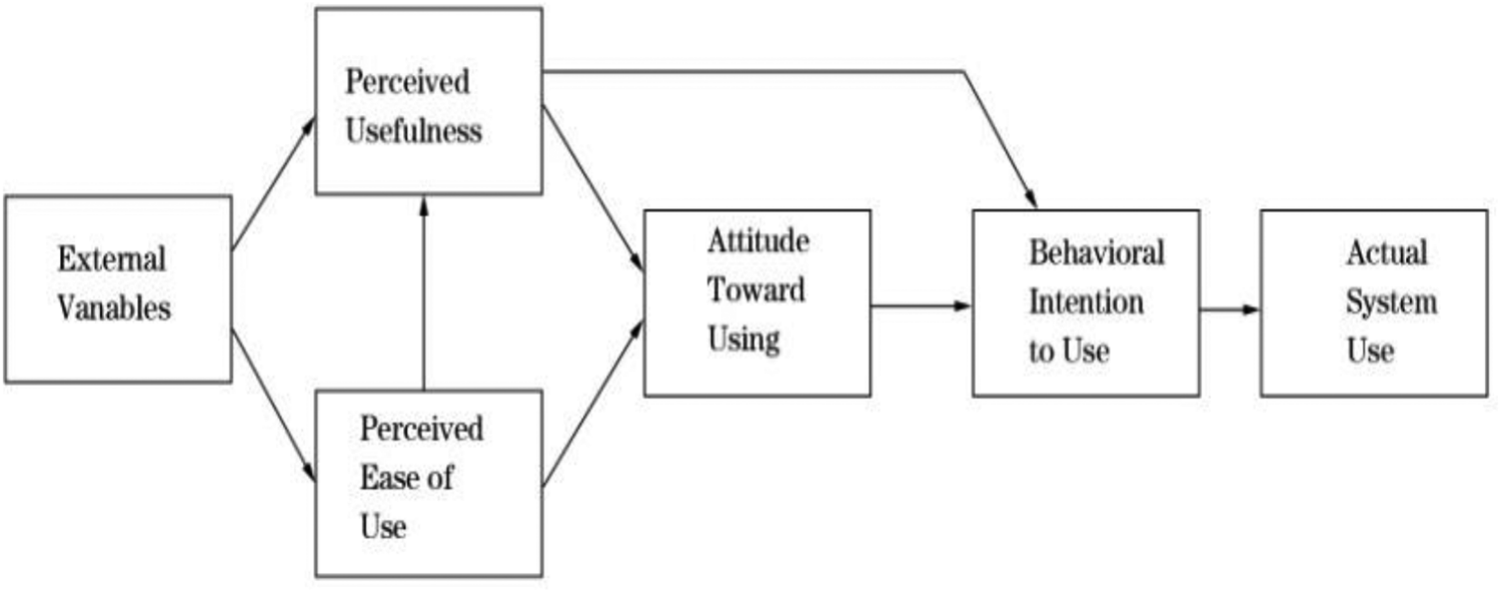
Technology acceptance model.

The Technology Acceptance Model (TAM) is an important theory in the field of information systems that aims to explain individuals' behavior in adopting new technologies (12). TAM focuses on the cognitive process of individuals toward new technologies, emphasizing the two core concepts of perceived usefulness and perceived ease of use (Figure 1). Previous studies have confirmed the effectiveness of TAM in explaining information system adoption behavior, including mobile applications and online services.

### 2.3 Self-perception theory

Self-perception theory is an important concept in social psychology, which describes how individuals form self-cognition by observing their own behavior and external feedback, thereby understanding their inner state and personality traits (13). Self-efficacy and privacy concerns are both forms of self-cognition. An individual's self-efficacy affects his or her behavior, thinking, and emotions. Individuals with high self-efficacy are more likely to take positive actions because they believe they can succeed, even in the face of challenges or difficulties. On the contrary, individuals with low self-efficacy may avoid challenges because they doubt their abilities (14). Privacy concerns refer to an individual's concerns and worries about personal information, privacy rights, and data security. In the digital age, as the digital collection, storage, and sharing of personal information become more and more common, people have become more concerned about the protection of personal privacy and data security (15). The higher the privacy concerns perceived by individuals, the lower the probability that they will use a new technology. On the contrary, they will be more likely to accept and use new technological products.

### 2.4 New information technology + mobile health management services system

In recent years, with the rapid development of the "Digital age" strategy, mobile health management services have attracted widespread attention from domestic and foreign scholars. Looking at domestic and foreign research, it is found that the research on new infrastructure + mobile health management services can be mainly divided into the following four categories: (1) Platform construction and service model of mobile health management services: Sun Wenxue (16) and others designed a mobile health management platform based on Bluetooth 4.0 technology and Android system. Mao Hui (17) and others built an overseas mobile health management platform based on the WeChat Mini Program. Wang Wenkai (18) and others proposed to build a health management information service platform in a digital publishing environment to monitor the health of users, help users develop a good lifestyle, and improve their health status. To achieve the professionalism and scientificity of health management information services, it provides support for improving the health information literacy and overall health level of the people. Yang Bin (19), Fan Chenhao (20), and Ren Xiaojia (21) used cloud platforms and big data to intelligently generate corresponding health management reminders for users' personal and surrounding environment information, thus realizing a panoramic and personalized intelligent health management model. (2) Application of emerging technologies in mobile management services: Caroline Free et al. (22), Lindquist AM et al. (23), and Bu Xiaofan (24) all agree that the use of virtual technology, digital technology, and information technology has the potential to change a person's psychological and physical condition, promote emotional regulation, relieve anxiety and depression symptoms, strengthen physical fitness management and weight management, improve bad habits, enhance people's health awareness and cognition of diseases, and improve health management services, (3) The current status and prospects of mobile health management services: Bruno M.C. Silva et al. (25), Zhao Dongxiang et al. (26) believe that mobile health management services are still in the early stages of exploration and are constantly trying to apply new mobile communication technologies, digital technologies, big data, and intelligent technologies in the health care field. Mobile health management services that incorporate new technologies have broader application prospects in developing countries and remote areas. (4) Acceptance of mobile health management services: Most scholars use the following research models and theories. For example, X. Guo, J (27), Hung M C (28), Sun Y (29), and Yang Zhao (30) respectively use the TAM model, comparative and integrated alternative model, motivation theory, protection motivation theory, planned behavior theory, innovation diffusion theory, Mate analysis, etc., hoping to explore the positive and negative factors of mobile health management service usage behavior from different perspectives and for different groups, such as those with a history of chronic diseases, sub-healthy people, and different age groups such as college students, young people, middle-aged people, and the older adult.

**Figure 2:**
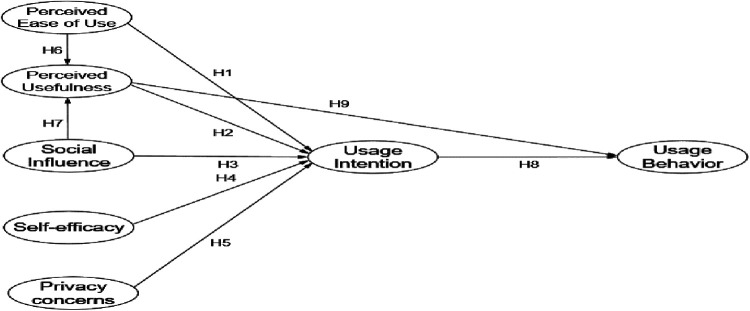
Research hypothesis model.

In summary, the purpose of the mobile health management service platform and various models currently built, designed and developed using new technologies is to more efficiently improve the health level of residents and reduce the medical and economic pressure on residents. The effectiveness needs to be verified in practice. The real feelings and feedback of users on the use of mobile health management services are the internal driving force for the high-quality development of mobile health management services. Based on this: This article proposes the following hypothesis, as shown in the hypothesis model diagram (Figure 2).

Hypothesis 1: Perceived ease of use is positively associated with intention to use.
Hypothesis 2: Perceived usefulness is positively associated with intention to use.
Hypothesis 3: Social influence is positively associated with intention to use.
Hypothesis 4: Self-efficacy is positively associated with intention to use.
Hypothesis 5: Privacy concerns are negatively associated with intention to use.
Hypothesis 6: Perceived ease of use is positively associated with perceived usefulness.
Hypothesis 7: Social influence is positively associated with perceived usefulness.
Hypothesis 8: Intention to use is positively associated with usage behavior.
Hypothesis 9: Perceived usefulness is positively associated with usage behavior.

## 3 Research design

### 3.1 Questionnaire design and variable measurement

This study follows a cross-sectional design and uses descriptive statistics and multiple linear regressions to explore the use behavior and influencing factors of mobile health management service systems for flight attendants in the context of the "Digital age". This paper adopts the questionnaire survey method to generate 7 latent variables according to the research model and research hypothesis of this paper. All latent variable measurement questions refer to the research results of predecessors and are designed in combination with the particularity of the mobile health management service system. Each measurement item is measured using the Likert 7-level scale, where 1 represents strongly disagree and 7 represents strongly agree. Then, the designed questionnaire was sent to 1 management expert, 1 health management behavior expert and 2 sports experts to evaluate the measurement tool and give opinions on the questionnaire. Then, the questionnaire was revised based on the measurement results and expert opinions of 56 flight crew members who were trained at the Civil Aviation Flight University of China to form the final questionnaire.

### 3.2 Data collection and statistical analysis

From June 2023 to September 2023, this study used the Wenjuxing platform to compile questionnaires and collect data. Considering the availability of samples, the study adopted a convenient sampling method due to logistical constraints, including limited access to flight attendants across multiple airlines and time restrictions during their irregular work schedules. This approach allowed efficient data collection within the three-month timeframe (June–September 2023). The managers of each airline issued questionnaires to the flight attendants of their company, and the survey was conducted in the form of online voluntary answers. A total of 456 questionnaires were collected. After careful screening of the questionnaires, 41 unqualified questionnaires were excluded, and the final number of valid questionnaires was 426. The effective rate was 93.44%; among the valid samples, in terms of the gender ratio of the subjects, males accounted for 56.8% and females accounted for 43.22%. The study procedures were approved by the Scientific Research Ethics Committee of Civil Aviation Flight University of China (No. 2023-05).

### 3.3 Data analysis methods

This study mainly used SPSS 24.0 and AMOS 24.0 software, and reliability and validity analysis were conducted using confirmatory factor analysis (CFA). Reliability was measured using Cronbach's *α* coefficient, and validity was measured using standard loading, composite reliability (CR), and average variance extracted (AVE); finally, the structural equation model (SEM) was used to analyze the overall model fit and path coefficients and to verify various research hypotheses. All data were collected through self-reported surveys, common method bias (CMB) is a concern. To assess CMB, Harman's single-factor test was conducted. The results indicated that no single factor accounted for the majority of variance. Nonetheless, potential CMB effects cannot be entirely ruled out; therefore, future research should incorporate alternative methods (e.g., marker variables or latent method factor approaches in SEM) to mitigate this issue.

## 4 Results of empirical analysis

### 4.1 Analysis of the current status of flight attendants' use of the health management service system

**Figure 3:**
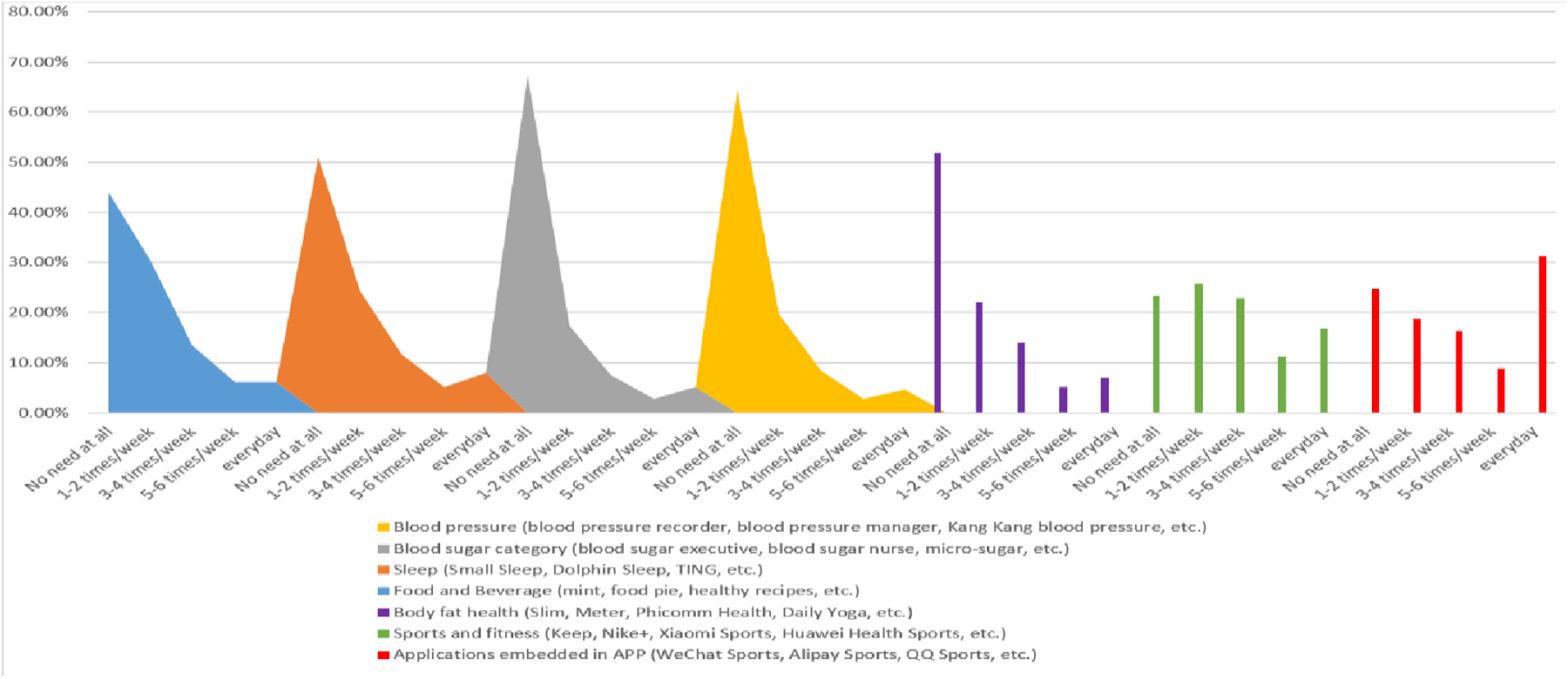
Usage of the flight attendant health management service system.

According to the data, in general, the utilization rate of the health management service system for flight attendants is not as high as expected. From Figure 3, it can be seen that the application usage of various mobile health management services (from completely not used to daily use) generally presents the same trend characteristics. More than half of the flight attendants in the survey do not use health service management apps such as blood sugar, blood pressure and body fat health. However, the use of sports and diet health management, sleep health management, body fat health and sports health monitoring applications is average among flight attendants. It is worth noting that sports and fitness applications and applications embedded in apps such as QQ Sports, WeChat Sports and Alipay Sports are very popular among flight attendants, among which the use rate of sports health monitoring applications reached 66.5%, and the daily frequency of use was as high as 25.52%. This shows that among the mobile health management service systems, the most used by flight attendants are those related to physical sports health.

### 4.2 Reliability analysis

Wu Minglong believes that Likert scale mainly uses split-half reliability or Cronbach α coefficient for reliability evaluation. Therefore, this study used SPSS24.0 software to analyze the reliability of the data and found that the Cronbach α coefficient of each measurement indicator was greater than 0.8 (see Table 2), which shows that the measurement indicators have good internal consistency and research reliability, and meet the reliability test requirements.

**Table 2:** Reliability analysis results.

Variables	Items	Cronbach’ a
Perceived ease of use	3	0.913
Perceived usefulness	3	0.910
Social influence	5	0.945
Privacy concerns	5	0.944
Self-efficacy	5	0.918
Usage intention	4	0.932
Usage behavior	3	0.909

### 4.3 Validity analysis

The measurement items of the scale in this study mainly draw on mature scales that have been verified by multiple tests at home and abroad and have been modified with reference to the opinions of relevant senior industry insiders. Therefore, the scale has good content validity. At the same time, discriminant validity and convergent validity can also be determined by factor analysis. Therefore, the questionnaire data was factor analyzed by SPSS24.0 software, and the total KMO value was 0.917, which is greater than 0.8, indicating that it is suitable for factor analysis. From Table 3, we can see that the loading coefficients of all observed variables on their latent variables are greater than 0.7, and the rotated component matrix is exactly 7 categories, which is consistent with the measurement model of the 7 dimensions constructed in this study. In summary, the measurement model of this paper has good validity. Therefore, this study can directly use confirmatory factor analysis to test the structural validity.

**Table 3:** Factor analysis rotated component matrix.

Factor	1	2	3	4	5	6	7
PU1	.104	−.057	.091	**.** **887**	.107	.103	.126
PU2	.107	−.080	.120	**.** **887**	.097	.121	.115
PU3	.116	−.075	.109	**.** **863**	.120	.145	.178
PE1	.199	−.150	.093	.179	.164	**.** **823**	.188
PE2	.153	−.160	.121	.112	.166	**.** **858**	.152
PE3	.136	−.167	.160	.127	.143	**.** **859**	.121
SI1	**.** **830**	−.201	.107	.029	.149	.139	.141
SI2	**.** **871**	−.125	.087	.109	.118	.137	.102
SI3	**.** **884**	−.142	.040	.079	.141	.089	.071
SI4	**.** **849**	−.193	.091	.111	.163	.091	.114
SI5	**.** **856**	−.132	.033	.089	.152	.095	.157
SE1	.252	−.140	.088	.149	**.** **842**	.144	.158
SE2	.197	−.128	.124	.126	**.** **838**	.173	.175
SE3	.219	−.095	.098	.093	**.** **862**	.160	.152
PC1	−.162	**.** **854**	−.065	−.020	−.084	−.113	−.125
PC2	−.208	**.** **860**	−.078	−.061	−.034	−.081	−.141
PC3	−.074	**.** **897**	−.086	−.077	−.073	−.103	−.083
PC4	−.174	**.** **864**	−.113	−.063	−.149	−.077	−.072
PC5	−.145	**.** **871**	−.090	−.052	−.065	−.141	−.102
UI1	.112	−.103	**.** **881**	.120	.070	.125	.074
UI2	.069	−.108	**.** **866**	.056	.100	.065	.187
UI3	.066	−.076	**.** **903**	.096	.032	.088	.111
UI4	.051	−.093	**.** **887**	.070	.098	.083	.096
UB1	.199	−.191	.194	.182	.172	.169	**.** **803**
UB2	.204	−.166	.154	.168	.157	.176	**.** **829**
UB3	.168	−.181	.208	.178	.214	.168	**.** **786**

Extraction method: principal component analysis. Rotation method: Kaiser normalization varimax. a. The rotation converged after 6 iterations.

#### 4.3.1 Convergent validity of the measurement model

The results in Table 4 show that the standardized loadings of each observed variable are all above 0.5 and have reached the significant level, indicating that the correlation between each dimension and the observed variables it constitutes is relatively high. The value of the latent variable combination reliability CR The range is 0.897–0.936, all higher than 0.7, and the range of AVE is 0.755–0.0.851, all higher than 0.5, indicating that the subordinate relationship between each latent variable and its constituent indicators is good, and each variable has good convergent validity.

**Table 4:** Convergent validity test of the measurement model.

Factors	Item	Parameter significance estimation				Std.	SMC	CR	AVE
		Unstd.	S.E.	*t*-value	*P*				
PU	PU1	1.000				.872	.760	.910	.772
	PU2	1.033	.044	23.660	***	.889	.790		
	PU3	.951	.041	23.183	***	.874	.764		
PE	PE1	1.000				.865	.748	.914	.779
	PE2	1.038	.043	23.983	***	.897	.805		
	PE3	.996	.042	23.655	***	.886	.785		
SE	SE1	1.000				.900	.764	.915	.782
	SE2	.880	.035	24.946	***	.874	.797		
	SE3	.938	.036	25.748	***	.893	.785		
PC	PC1	1.000				.858	.736	.944	.772
	PC2	1.053	.044	24.145	***	.874	.764		
	PC3	1.071	.042	25.263	***	.895	.801		
	PC4	.998	.041	24.563	***	.882	.778		
	PC5	1.027	.042	24.608	***	.883	.780		
SI	SI1	1.000				.861	.741	.945	.776
	SI2	1.031	.041	25.366	***	.892	.796		
	SI3	1.050	.041	25.412	***	.893	.797		
	SI4	1.024	.041	25.020	***	.886	.785		
	SI5	1.052	.043	24.252	***	.871	.759		
UB	UB1	1.000				.885	.783	.910	.771
	UB2	1.023	.042	24.301	***	.895	.801		
	UB3	.992	.043	22.880	***	.853	.728		
UI	UI1	1.000				.885	.783	.932	.776
	UI2	.955	.039	24.614	***	.862	.743		
	UI3	1.063	.039	26.968	***	.903	.815		
	UI4	.927	.037	25.172	***	.872	.760		

*p* < 0.05, *p < 0.01 (34).

*** *p* < 0.001.

#### 4.3.2 Measurement model discriminant validity

The discriminant validity between variables is mainly measured by comparing the correlation coefficient between the variables with their AVE square root. If the AVE square root of the variable is greater than the correlation coefficient of other related facets, it means that the variable is discriminable from other variables. As can be seen from Table 5, the AVE square root of each variable is greater than the correlation coefficient of the facet, indicating that the variables in this study have good discriminant validity.

**Table 5:** Discriminant validity table.

	AVE	UB	UI	PC	SE	SI	PE	PU
UB	.771	**.** **878**						
UI	.776	.426	**.** **881**					
PC	.772	−.422	−.261	**.** **879**				
SE	.782	.529	.284	−.329	**.** **884**			
SI	.776	.463	.236	−.402	.495	**.** **881**		
PE	.779	.520	.329	−.383	.480	.408	**.** **883**	
PU	.772	.467	.286	−.224	.366	.300	.395	**.** **879**

### 4.4 Model fit analysis

This study uses AMOS24.0 software to model the usage behavior of the flight attendant health management service system, and obtains a structural equation model consisting of 7 variables and 28 measurement indicators through maximum likelihood estimation method analysis. At the same time, considering that there are many fitting indicators for structural equation models, this article uses some common indicators to analyze the fitting degree of the sample, and the model fitting index is obtained as follows: CMIN(*χ*2) = 429.020, df = 284, CMIN (*χ*2)/df = 1.511, GFI = 0.931, AGFI = 0.914, CFI = 0.985, NNFI(TLI) = 0.983, RMSEA = 0.035, IFI = 0.985. In statistics, scholars believe that (*χ*2/df) < 3, RMSEA < 0.08, GFI, NNFI, IFI > , CFI > 0.9, and AGFI > 0.8 are statistically significant. The indicators of the structural equation model in this study are intended to be statistically significant. The degree of fit is high and in line with social statistical measurement standards, indicating that the overall structure of the model is reasonable.

### 4.5 Model path analysis

**Figure 4:**
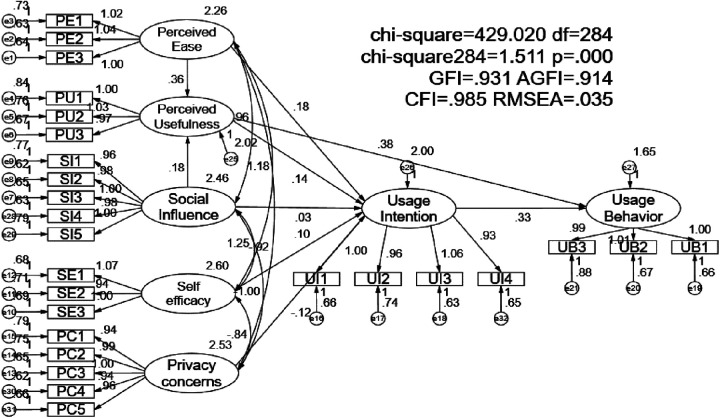
Test results of the integrated structural equation model (standardized solution).

This study tested the impact of each aspect in the model, performed path testing on the model, and adopted standardized effect values to obtain the model path coefficients and path testing table (Table 6) (Figure 4). The results show that except for the standardized path coefficient of H5, which is negative, the standardized path coefficients of H1 to H9 are all higher than 0. When the absolute value of CR is greater than 1.96 and the *p*-value is less than 0.05, it can be determined that the regression coefficient of the hypothesized path is significant. When the path coefficient appears negative, it indicates that there may be a reverse correlation in the relationship between variables. According to this standard, according to the results in Table 6, the *p*-values of H1 and H2 are less than 0.05, the results are significant, and the hypothesis is passed. The *p*-values of H3 and H4 are greater than 0.05, the results are not significant, and the hypothesis fails. The *p*-value of H5 is less than 0.05, and the result is significant. However, the path coefficient of H5 is negative, indicating that there is a negative correlation between privacy concerns and usage intention. The hypothesis passes. The *p*-values of H6-H9 are less than 0.001, the results are very significant, and the hypothesis passes.

**Table 6:** Model path test results.

Hypothesis	Path relationship	Path coefficient	Standard error	C.R.	*P*	Label	Result
H1	PE → UI	.175	.065	2.675	.007	H1	Pass
H2	PU → UI	.135	.054	2.501	.012	H2	Pass
H3	SI → UI	.028	.059	.479	.632	H3	Fail
H4	SE → UI	.101	.059	1.702	.089	H4	Fail
H5	PC → UI	−.121	.054	−2.264	.024	H5	Pass
H6	PE → PU	.361	.058	6.198	***	H6	Pass
H7	SI → PU	.181	.054	3.348	***	H7	Pass
H8	UI → UB	.329	.049	6.758	***	H8	Pass
H9	PU → UB	.384	.049	7.907	***	H9	Pass

*p* < 0.05, *p < 0.01 (34).

*** *p* < 0.001.

## 5 Discussion and analysis

The data analysis results of this paper reveal the specific situation of flight attendants' use of mobile health management services under the background of "Digital age", and have a deeper understanding of the mechanism affecting mobile health management services. It has important implications and reference significance for the upgrade, optimization and management practice of mobile health management service systems.

### 5.1 The impact of perceived ease of use (PE) on usage intention (UI)

Perceived ease of use significantly and positively affects intention to use, indicating that if flight attendants believe that the mobile health management system is easy to use, they are more likely to intend to use it. This finding supports the core idea of the Technology Acceptance Model (TAM), that is, ease of use is one of the key factors when users evaluate a new technology (12). In the working environment of flight attendants, where time and energy are limited, the ease of use of the system becomes particularly important. In addition, a system with high ease of use can reduce the time for learning and adaptation, allowing users to become familiar with and use the system more quickly, thereby improving work efficiency.

### 5.2 The impact of perceived usefulness (PU) on usage intention (UI)

Perceived usefulness significantly and positively affects intention to use, which is consistent with TAM expectations, that is, if users believe that a system is useful, they are more likely to intend to use it (12). In a high-stress and highly mobile work environment, flight attendants are more willing to use the mobile health management system if they believe that it can effectively help them manage their health. The mobile health management system can provide real-time health monitoring, health advice, and emergency medical support, which are very important for flight attendants' health management. Therefore, its perceived usefulness directly affects their intention to use the system.

### 5.3 Impact of social influence (SI) on usage intention (UI)

Social influence has no significant effect on intention to use, which may be because the work environment and social network of flight attendants are relatively special, and they rely more on personal judgment rather than social recognition. In high-stress occupations, individuals may pay more attention to the practical utility and ease of use of technology rather than the opinions of other colleagues (31). In addition, the characteristics of flight attendants' work determine that they have less time to communicate and interact with their colleagues, so social influence has less impact on their technology usage decisions. The non-significant effect of social influence (*β* = 0.028, *p* = 0.632) contrasts with UTAUT predictions (Venkatesh et al., 2003). This divergence may stem from the decentralized nature of flight attendants' work, where peer interaction is limited compared to office-based professions (Hung & Jen, 2010). Unlike nurses or teachers, flight attendants operate in transient teams, reducing opportunities for peer-driven adoption. However, social influence positively impacted perceived usefulness (*β* = 0.181, *p* < 0.001), suggesting institutional endorsements (e.g., airline management promoting MHMS) could indirectly foster uptake.

### 5.4 The impact of self-efficacy (SE) on usage intention (UI)

The insignificant effect of self-efficacy on intention to use may be related to the professional training and high standards of self-requirements of flight attendants. They may already have high self-efficacy, so when using new technologies, perceived usefulness and ease of use have a greater impact on them (32). In addition, self-efficacy may be considered a capability that is taken for granted in this context, rather than a key factor affecting intention to use technology.

### 5.5 Impact of privacy concerns (PC) on usage intentions (UI)

Privacy concerns significantly negatively affect usage intention, indicating that flight attendants are very concerned about the security of their personal information when using mobile health management systems. This result is consistent with previous studies that privacy concerns are one of the important barriers to technology adoption (33). Flight attendants' privacy sensitivity (*β* = −0.121, *p* < 0.05) aligns with aviation regulations requiring strict health monitoring (Zhao et al., 2018). For instance, fitness-to-fly assessments could lead to employment repercussions if data is mishandled. To increase usage intention, system developers need to ensure and communicate the privacy protection measures of the system to users. Privacy issues are particularly sensitive for flight attendants because their health data may involve important aspects such as occupational health and flight safety. Mitigation Strategies: such as implement end-to-end encryption (e.g., blockchain-based systems; Yang et al., 2022), provide granular privacy controls (e.g., opt-in sharing for specific health metrics), and Adopt GDPR-inspired anonymization techniques to decouple personal identifiers from health data (Smith et al., 2011).

### 5.6 Effect of perceived ease of use (PE) on perceived usefulness (PU)

Perceived ease of use significantly and positively affects perceived usefulness, indicating that systems that are easy to use are considered more useful. This is consistent with the extended model of TAM, where ease of use not only directly affects intention to use, but also indirectly affects it through perceived usefulness. When flight attendants find the system easy to operate, they are more likely to recognize the actual value of the system in health management, thereby improving their perception of the system's usefulness.

### 5.7 Effect of social influence (SI) on perceived usefulness (PU)

Social influence has a significant positive impact on perceived usefulness, which may be because when colleagues or superiors approve of the system, individuals are more likely to think that the system is useful. This result supports the unified theory (UTAUT), which states that social influence can indirectly affect usage behavior by improving perceived usefulness. In airlines, if leaders and colleagues actively promote and use the health management system, individuals are more likely to accept and think that the system helps improve work efficiency and health management results.

### 5.8 The influence of usage intention (UI) on usage behavior (UB)

The usage intention significantly and positively affects the usage behavior, which is consistent with Ajzen's (1991) theory of planned behavior, where intention is the direct antecedent of behavior. Strong usage intention will be converted into actual usage behavior. This means that once flight attendants decide to use the mobile health management system, they are likely to put it into practice and actually adopt the system to manage their health.

### 5.9 The influence of perceived usefulness (PU) on usage behavior (UB)

Perceived usefulness significantly and positively affects usage behavior, further confirming the key role of usefulness in technology adoption. The more useful a user thinks a system is, the more likely they are to use it. For flight attendants, if they believe that the mobile health management system can effectively improve health and work performance, they will be more willing and motivated to use the system. They will be more motivated to use it.

## 6 Summary and outlook

This study explored the effects of perceived ease of use, perceived usefulness, social influence, self-efficacy, and privacy concerns on the use of mobile health management service systems by flight attendants. The results showed that perceived ease of use significantly and positively affected the intention to use and perceived usefulness, verifying the validity of the Technology Acceptance Model (TAM). Perceived usefulness had a significant positive impact on both the intention to use and the behavior to use, further confirming the key role of usefulness in technology adoption. Social influence had no significant effect on the intention to use, but had a significant positive effect on perceived usefulness, indicating that social influence indirectly affected the behavior to use through perceived usefulness. Self-efficacy had no significant effect on the intention to use, probably because the professionalism and self-efficacy levels of flight attendants were generally high. Privacy concerns significantly and negatively affected the intention to use, showing the importance of privacy issues in the adoption of health management systems. The intention to use significantly and positively affected the behavior to use, which was consistent with the theory of planned behavior. These findings not only expanded the application of TAM in specific occupational groups, but also provided practical guidance for the design and promotion of mobile health management systems. It should be noted that this study also has certain limitations. First, in terms of the selection of research samples, the sample sampling adopted the method of network convenience sampling, this method may introduce selection bias, as participants were volunteers from airlines collaborating with the research team, potentially underrepresenting flight attendants from smaller or non-cooperative airlines, future studies should employ stratified or random sampling to enhance representativity and its sampling range and sampling quantity were not sufficient, which may have certain limitations in universality; secondly, the mediating relationship between several variables in the constructed new model was not taken into account, and no hypothesis and analytical test of the mediation effect was performed. Future research can be further deepened from the following aspects: Diversified samples: This study focuses on flight attendants, and can be expanded to other occupational groups in the future to verify the universality and diversity of the results. The working environment and health management needs of different occupational groups may affect the determinants of system usage behavior. Long-term longitudinal research: A longitudinal research design is used to track changes in user behavior in using the system to further understand the long-term effects of influencing factors. Intervention measures for privacy concerns: In-depth exploration of how to effectively alleviate users' privacy concerns, such as enhancing data security through technical means, or enhancing users' confidence in privacy protection measures through education. Privacy concerns are a complex issue and may need to be addressed from multiple levels, including technology, policy, and user education. Impact of technological advances: With the rapid development of technology, the functionality and user experience of mobile health management systems are constantly improving. Research needs to keep up with the latest technological changes and explore their impact on user perception and usage behavior. New technologies such as artificial intelligence and big data analysis may significantly change the ease of use and usefulness of the system. Through further research, we can have a more comprehensive understanding of the usage behavior of mobile health management systems in different scenarios, thereby optimizing system design and improving user acceptance and usage effects. This will not only help improve the health management level of flight attendants, but also provide valuable health management tools for other occupational groups and the general public. In future development, the combination of technology, privacy protection, and user education will be the key to promoting the widespread application of mobile health management systems.

## Data Availability

The datasets presented in this article are not readily available because additional use of the dataset may require approval from the original creators or custodians of the data. Requests to access the datasets should be directed to lyt9027@cafuc.edu.cn.
